# Application evaluation of clinical practice guidelines for traditional Chinese medicine: a clinical analysis based on the analytic hierarchy process

**DOI:** 10.1186/s12906-019-2683-5

**Published:** 2019-10-22

**Authors:** Huayang Cai, Hui Li, Huizhen Zeng, Danping Xu, Wenwei Ouyang, Aiping Lv

**Affiliations:** 1 The Second Clinical College of Guangzhou University of Chinese Medicine(Guangdong Provincial Hospital of Chinese Medicine), No.111 Road Dade, Guangzhou, China; 2 Hong Kong Baptist University, Hong Kong, China

**Keywords:** Clinical practice guidelines, Traditional Chinese medicine, Application evaluation, Angina pectoris, Analytic hierarchy process

## Abstract

**Background:**

Clinical Practice Guidelines (CPGs) play an important role in clinical practice, and they require appropriate evaluation, especially in application. This study explores the application evaluation method of CPGs for Traditional Chinese Medicines (TCM). It uses the Analytic Hierarchy Process (AHP) and clinical cases to evaluate the consistency between CPGs of TCM and clinical practice.

**Methods:**

To evaluate the consistency between CPGs of TCM and clinical cases, a 3-level AHP construction was built. Weightings were calculated by collecting questionnaires according to AHP theory. To test the evaluation system, a retrospective study was performed. The study evaluated the China Association of Chinese Medicine’s Guidelines for Diagnosis and Treatment of Common Internal Diseases in Chinese Medicine Diseases of Modern Medicine (CPGs of DTCID) (ZYYXH/T50–135-2008). A total of 150 cases were involved. The evaluation system was used to assess the consistency between CPGs of DTCID and clinical cases of angina pectoris.

**Results:**

The results showed that the overall consistency between CPGs of DTCID and the 150 cases was 42.32 ± 6.94%, ranging from 35.21 to 63.37%. The overall consistency was not affected by age, gender, type of angina pectoris, condition of percutaneous coronary intervention (PCI), or angina classification as determined by the Canadian Cardiovascular Society. The consistencies of each index were as follows: Diagnosis of TCM, 100%; Diagnosis of Western medicine, 100%; Syndrome classification, 38.25 ± 4.40%; Syndrome key point, 34.17 ± 8.15%; TCM Decoction, 31.08 ± 23.64%; TCM particular treatment, 7.92 ± 19.13%; and Recuperation and prevention, 0. The most frequent syndromes were *qi*-deficiency, phlegm and blood stasis (*n* = 124) (82.7%). The overall consistency of *qi*-deficiency, turbid phlegm and blood stasis was lower than the overall consistency of the group without that syndrome. The difference was statistically significant (*P* < 0.05). 42 cases (28%) applied the TCM decoction recommended by CPGs of DTCID. Of these, *Gualouxiebaibanxia* decoction was applied in 34 cases. *Wendan* decoction, the most frequently used, was applied in 64 cases (42.7%).

**Conclusion:**

This study indicates that the AHP system can perform quantitative evaluation of consistency between TCM CPG and clinical practice. It also found the factors affecting the application of TCM CPGs and might indicate the need for revisions of CPGs.

## Background

The clinical practice guidelines (CPGs) used to assist clinicians and patients in making healthcare decisions play an important role in clinical practice [1]. Even systematically developed CPGs require proper evaluation for several reasons including inferior methodology, inapplicability to clinical practice, or the inconsistent recommendations from different CPGs may give for a clinical problem [2, 3]. The proliferation of Traditional Chinese Medicine (TCM) has necessitated proper evaluation, as would be the case for any other type of CPGs. Many CPGs evaluation instruments have been developed, and the Appraisal of Guidelines, Research and Evaluation (AGREE) has been applied to a wide range of them. AGREE was developed in 2003 by researchers from 13 countries. Version 2, which came out in 2009 focused on methodological quality, including evaluative dimensions for scope and purpose, stakeholder, involvement, rigour of development, clarity of presentation, applicability and editorial independence [4]. Although widely used, AGREE is not ideal for evaluating the application of CPGs [5, 6]. Application is important for CPGs because without application, CPGs have less value for clinicians.

TCM has been evolving for thousands of years, and is dependent on clinical experience. Its advantage is individualized diagnosis and treatment, but at present TCM lacks the high-level clinical evidence essential for CPGs of high methodological quality [7]. Using AGREE to evaluate the CPGs of TCM may produce the misleading conclusion that the quality of CPGs for TCM is unsatisfactory. Therefore, an evaluation method for CPGs of TCM application is needed.

To evaluate the application of CPGs, we have adopted a comprehensive evaluation method to appraise the consistency between CPGs of TCM and clinical practice. We have built a consistency evaluation system (heretofore “the system”) using the Analytic Hierarchy Process (AHP). AHP is a common comprehensive assessment [8]. In this study, we have used clinical cases to calculate the consistency of CPGs for TCM and clinical practice, using AHP.

## Methods

To build the system, we used AHP theory, and calculated the weight of indexes by consulting clinicians according to the Saaty weight method.

To test the system based on the clinical cases, we used the China Association of Chinese Medicine’s Guidelines for Diagnosis and Treatment of Common Internal Diseases in Chinese Medicine Diseases of Modern Medicine (CPGs of DTCID) (ZYYXH/T50–135-2008) [9]. We then conducted a retrospective quantitative analysis of the consistency between CPGs of DTCID for angina and 150 angina cases.

### Constructing the system

#### System structure

**Fig. 1:**
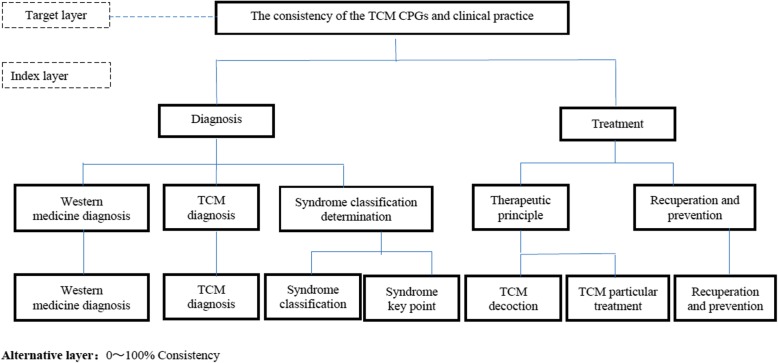
Construction and weighting of the system

According to AHP theory, the system should be composed of 3 layers: target layer, index layer and alternative layer. According to CPGs of DTCID and the preliminary research foundation, the index layer was broken into 3 level including 7 indexes (see Fig. 1) These 7 indexes in Diagnosis and Treatment, which were considered as important and basic factors included in CPGs of TCM might display the characteristics of TCM. On the basis of this idea, these 7 indexes were also referred to the following information: Application Evaluation Questionnaire Formed by the 2012 Public Health Special Fund for GPGs of TCM Application Evaluation Project by State Administration of Traditional Chinese Medicine, the content of CPGs of DTCID, and the content of Textbooks of TCM internal medicine.

Therefore, the AHP evaluation system structure was constructed by 3 level including 7 indexes.

#### Questionnaire consultation

The purpose of this study was to assess the consistency between the CPGs of TCM and clinical practice. Participants in TCM standard application (May 17–18, 2014) and training courses for TCM CPGs application (July 19–20, 2014) hosted by Guangdong provincial hospital of Chinese Medicine, who were Chinese medicine practitioners as real users of the CPGs in practice considered suitable to be consulted.

In this study, according to the requirements of the analytic hierarchy method, (Fig. 1) a consultation questionnaire (Additional file 1) was established. To obtain the comparison of the indexes of the various levels, questionnaires were distributed in two training courses mentioned above. Consultants compared the relative importance of indexes in the same level, for example of Diagnosis and Treatment, Syndrome classification and Syndrome key point, TCM decoction and TCM particular treatment. Etc.

#### Weight calculation [8]

##### Matrix determination

For the indexes in the primary, secondary and tertiary levels, a pairwise comparison matrix was established.

A was the pairwise comparison matrix of the primary indexes diagnosis and treatment, which was used to evaluate the importance weight of these two indexes.

$$ A=\left[\begin{array}{cc}a11& a12\\ {}a21& a22\end{array}\right] $$

B was the pairwise comparison matrix of the secondary indexes diagnosis, which was the comparison of TCM diagnosis, Western medicine diagnosis and syndrome differentiation and used to evaluate the importance weight of these three indexes.

$$ B=\left[\begin{array}{ccc}b11& b12& b13\\ {}b21& b22& b23\\ {}b31& b32& b33\end{array}\right] $$

Also we built matrix C for Treatment, matrix D for syndrome differentiation determining and matrix E for therapeutic principle.

**Score determination by the Saaty method**

According to the Saaty weighting method, for the index *i* and the index *j* in the same matrix, we compared the importance of between them relative to the previous level and used the quantized relative weight a_ij_ to describe. It was called the pairwise comparison matrix in which a total of *n* indexes participating in the comparison.

The value of a_ij_ is between 1 and 9 and its reciprocal.

• a_ij_ = 1, index i and index j have the same importance to the previous level.

• a_ij_ = 3, index i is slightly more important than index j.

• a_ij_ = 5, index i is more important than index j.

• a_ij_ = 7, index i is much more important than index j.

• a_ij_ = 9, index i is extremely important than index j.

(2, 4, 6, 8) was the intermediate value of the two adjacent degrees and was used when necessary.

The values of b_ij_, c_ij_, d_ij_, and e_ij_ were the same as above.

##### Weight calculation

Based on each valid consultation questionnaire we calculated weightings of AHP systems so that we could get the average score of each index. The detail were as follow:

##### Step 1 calculating the weight coefficients *w*

We calculated the average score of the consultations of a_ij_, b_ij_, c_ij_, d_ij_, and e_ij_ in Matrix A, B, C, D, and E.

Then we calculated the initial weight coefficient *w*_*i*_' of each index using the average score and the formulate as follow:

$$ {w}_i`=\sqrt[m]{a_{i1}\bullet {a}_{i2}\cdots {a}_{im}} $$

So we calculated the normalized weight coefficient *w* of each index using the formulate as follow:

$$ w={w}_i`/\sum \limits_{i=1}^mw{`}_i $$

##### Step 2 calculating the indicator CI

The indicator CI which measured the degree of inconsistency of a pairwise comparison matrix A, was calculated using the formulate as follow:

$$ \mathrm{CI}=\frac{\lambda_{\mathrm{max}}\left(\mathrm{A}\right)-\mathrm{n}}{n-1} $$

$$ {\lambda}_{\mathrm{max}}=\sum \limits_{i=1}^n{\lambda}_i/n,{\lambda}_i=\sum \limits_{j=1}^n{a}_{ij}{w}_j/{w}_i $$

*n* is the number of sub-indexes of the tested level. *λ*_max_ is maximum eigenvalue. *λ*_*i*_ is the eigenvalue of pairwise compared sub-indexes in the preferred matrix of the level.

##### Step 3 calculating the indicator RI and CR

RI was the average random consistency indicator, and the value was related to the matrix order *n*. The standard RI for checking the consistency of pairwise comparison matrices is as follows [10]. (see Table 1).

**Table 1:** The value of RI of the 1–9 order average random consistency index

n	1	2	3	4	5	6	7	8	9
RI	0	0	0.58	0.90	1.12	1.24	1.32	1.41	1.45

We calculated the random consistency ratio (CR)of the pairwise comparison matrix according to the following formula:

$$ \mathrm{CR}=\frac{CI}{RI} $$

The judgment method was as follows: When CR < 0.1, it was determined that the pairwise comparison matrix had satisfactory consistency, or the degree of inconsistency was acceptable. Otherwise, the pairwise comparison matrix needed to be adjusted until satisfactory consistency was achieved.

The above calculation process was performed via the formula function in Microsoft Excel 2011. We set up the evaluation system with the help of v7.5, a yaahp AHP software package developed by Beijing Xing Cheng Software Technology, Co., Ltd.

#### Testing the system based on the clinical cases

The Testing of the system based on the clinical cases was performed in the general way as follow:the evaluation group checked the clinical case met the diagnosis, compare the clinical diagnosis and treatment of the hospital and the suggestion of the CPGs on each index and determined the score of each index. Then the system would calculate the consistency of the single clinical case so that the general situation of 150 case was available.

#### The evaluation of CPG of TCM

CPGs of DTCID for angina pectoris were selected to be evaluated by the system.

#### Cases used for the system testing

To test the system, we performed a retrospective study involving 150 cases which had met the diagnostic of CPG of DTCID for angina pectoris and had been hospitalized on April 21, 2014 or later.

#### Evaluation method

In this retrospective study, we created a group which evaluated the consistency between the CPG of DTCID and clinical practice among 150 angina cases using the questionnaire (Additional file 2) as the basis of the system.

#### Comparison object conversion

The fundamental element of the AHP method is pair-wise comparison between object A and object B (e.g. two treatment programs or two material objects). The comparison begins with AHP Saaty weighting method (scored 1/9 to 1 or 1 to 9). For a certain index, a score of 9 means that object A is extremely important compared to object B, while a score of 1/9 means that object B is extremely important. 1 means objects A and B are equally important. To evaluate the consistency of CPGs and clinical practice, we converted object A and object B to minimum (100% inconsistent) and maximum (100% consistent) degrees of consistency.

#### Questionnaire design

According to the requirements of the AHP and the standards of the Saaty weight method, we converted the Saaty weightings from comparison of relative importance to a 0–100% consistency degree. These degrees were based on the CPG evaluated and the case involved (see Table 2).

**Table 2:** Traditional AHP Saaty weight method, and with conversion to consistency evaluation

Traditional AHP Saaty Scale		Converted to consistency evaluation	
Importance on scale	Definition	Consistency degree between CPG and Clinical Practice(%)	Score in the system
1	Compared to B, A is of equal importance	50	1
3	Compared to B, A is slightly more important	62.5	3
5	Compared to B, A is more important	75	5
7	Compared to B, A is much more important	87.5	7
9	Compared to B, A is extremely important	100	9
2, 4, 6, 8	Compared to B, A is important with an intermediate value between the two judgments	–	2, 4, 6, 8
1/3	Compared to A, B is slightly more important	37.5	1/3
1/5	Compared to A, B is more important	25	1/5
1/7	Compared to A, B is much more important	12.5	1/7
1/9	Compared to A, B is extremely important	0	1/9
1/2,1/4,1/6,1/8	Compared to A, B is important with intermediate values between the two judgments	–	1/2,1/4,1/6,1/8

#### Establishment of the assessment group

The evaluation group consisted of Dr. Danping Xu (associate chief physician of the cardiology center) and Dr. Huayang Cai (associate chief physician, leader of this study). After discussing each clinical case in every index of the system, the group determined its score.

#### Principles of evaluation

The principles of 7 indexes were followed to determine a score.

TCM diagnosis and Western medicine diagnosis index: If all included cases met the CPG diagnosis criteria, “totally consistent” (100%) was marked.

Syndrome classification index: The group divided the clinical case syndrome and guidelines’ syndrome into several syndrome factors, and discussed the consistency percentages.

Syndrome key point index: The clinical case symptoms with symptoms in the guidelines so as to discuss and determine the consistency percentage.

TCM decoction index: The TCM decoctions between the cases and the CPG were compared, and the group determined the consistency percentage. If there were no TCM decoctions in the case, they were scored as “totally inconsistent” (0%).

TCM particular treatment((Non TCM decoction))index: A particular TCM therapy was compared between the cases and the CPGs, and the group determined the consistency percentage. If the cases had refused a particular TCM therapy, they were scored as “totally inconsistent” (0%).

Recuperation and prevention indexes: The content about recuperation and prevention was compared between the cases and the CPG, and the group determined the consistency percentage. lf there was no content in either the case or in the CPG, they were scored as “totally inconsistent” (0%).

#### Evaluation standards for clinical effects and safety

The Canadian Cardiovascular Society (CCS) angina severity classification was applied to evaluate the clinical effects. The WHO International Drug Monitoring Cooperation Center definitions of adverse drug reactions (ADR) were applied to evaluate safety.

#### Sample size estimation

In this study we calculated the sample size by Kappa Test for Agreement Between Two Raters with 90% power to detect a true Kappa value of 0.80. A sample size of 113 was the result from the calculation while actually 150 cases met the result.

#### Data management and statistical analysis

PASW Statistics 18.0 was used to enter questionnaire data, build the database and perform statistical analysis. The software yaahp AHP v7.5 developed by Foreology Software Ltd. in Beijing, was used to construct the evaluation system and calculate each case’s consistency degree in line with the principle of AHP.

By using PASW Statistics 18.0, descriptive statistics of the included cases was performed; the comparison of consistency degree between the groups was performed by Rank Sum Test; the relationship between the consistency degree and efficacy was analyzed by Correlation Analysis; the count data were analyzed by chi-square test, *p* = 0.05.

#### Ethics approval and consent to participate

For this study was performed as a retrospective clinical study and the information of clinical cases was collected form the cases database in the computer, we applied for exemption from ethical review(Application Number B2013–175) before the study began in 2013.

In compliance to the Good Clinical Practice for Clinical Trial of New Drugs, Guidelines for Ethics Review of Drugs Clinical trial (approved by China Food and Drug Administration), Ethics Review for Biomedical Research Involving Human Subjects, Management of Clinical Research on Stem Cells (for Trial Implementation) (issued by Ministry of Public Health of China), Standards for Ethics Review Platform Construction of Chinese Medical Research (issued by State Administration of Traditional Chinese Medicine), Declaration of Helsinki, International Ethical Guideline for Biomedical Research involving Human Subjects, the Ethics Committee of Guangdong Provincial Hospital of Chinese Medicine agrees the exemption from Review.

## Results

### Weightings of indexes and the evaluation system

**Fig. 2:**
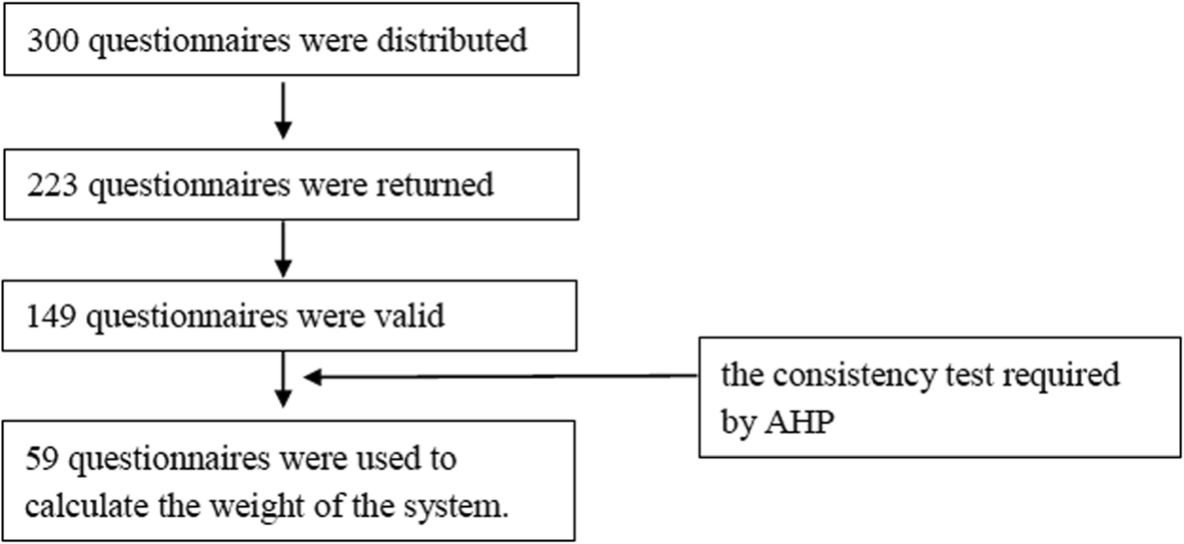
System Questionnaires. AHP theory requires matrix consistency testing to exclude logic errors in questionnaires. This matrix consistency testing was performed, which 59 questionnaires passed and used to calculate the weight of the system. This matrix consistency testing which is one step of building AHP construction, is not the concept as testing the consistency between the CPGs and clinical practice

We consulted clinicians with regards to the Saaty weighting method. 59 questionnaires passed the consistency measurement required by AHP (see Fig. 2), and the weightings of 7 indexes were calculated to determine the system(Table 3).

**Table 3:** The weight of each index

Index layer(level 3)	level 3 weight	Index layer (level 2)	level 2 weight	Index layer (level 1)	level 1 weight	Total
Western medicine diagnosis	0.13214	Western medicine diagnosis	0.13214	Diagnosis	0.553124	1
TCM diagnosis	0.163026	TCM diagnosis	0.163026			
Syndrome classification	0.115915	Syndrome classification determination	0.257958			
Syndrome key point	0.14203					
TCM decoction	0.21166	therapeutic principle	0.290852	treatment	0.44931	
TCM particular treatment	0.079236					
Recuperation and prevention	0.158458	recuperation and prevention	0.158458			

The age of the consultants included in the calculation was 36.98 ± 7.212 years; the years of experience was 12.32 ± 8.365. There were 39 males and 20 females.

### 150 Angina pectoris cases and its overall consistency evaluation

The overall consistency degree of the 150 angina inpatients was 42.32 ± 6.94%, and the range was from 35.21 to 63.37%(Table 4).

**Table 4:** Basic biographical information for the 150 angina inpatients and overall consistency comparison

Category		Number(Ratio)	consistency degree(%)	*z*	*P*
Gender (number)	male (%)	77(51.33)	41.92 ± 6.66	−0.722	0.471
	female (%)	73 (48.67)	42.74 ± 7.24		
Age	age (year)	68.43 ± 11.45			
Hospital stay	hospital stay (day)	7.05 ± 2.53			
Western medicine diagnosis (number)	stable angina (%)	85 (56.67)	42.58 ± 7.02	0.523	0.602
	unstable angina (%)	65 (43.33)	41.98 ± 6.87		
PCI operation (number)	PCI surgery before hospitalization (%)	39 (26.00)	42.68 ± 6.99	0.207	0.814
	PCI surgery during hospitalization (%)	41 (27.33)	41.74 ± 6.26		
	no PCI surgery (%)	70 (46.67)	42.45 ± 6.94		
CCS classification	class 1	9	44.28 ± 8.52	0.800	0.496
	class 2	54	41.25 ± 5.98		
	class 3	26	42.56 ± 6.59		
	class 4	61	42.87 ± 7.63		

Table 4 shows that basic biographical information of 150 cases and there was no statistically significant correlation between factors such as gender, age, PCI surgery, CCS classification and the overall consistency of the 150 cases and CPGs of DTCID.

### Consistency, based on each tertiary index

Table 5 shows that the index consistency for TCM syndromes and treatments with the most weight is low. The enrolled cases each passed the diagnosis requirements of the CPG, and thus the indexes came to 100%. The consistency of recuperation and prevention was 0%. This is the result of content not mentioned in the CPG.

**Table 5:** Consistency of each tertiary index

Tertiary index	minimum	maximum	consistency (%)
TCM diagnosis (%)	100.00	100.00	100.00 ± 0.00
Western medicine diagnosis (%)	100.00	100.00	100.00 ± 0.00
Syndrome classification (%)	25.00	50.00	38.25 ± 4.40
Syndrome key point (%)	12.50	62.50	34.17 ± 8.15
TCM decoction (%)	0.00	100.00	31.08 ± 23.64
TCM particular treatment(%)	0.00	87.50	7.92 ± 19.13
Recuperation and prevention (%)	0.00	0.00	0.00 ± 0.00

### Syndrome classification and dialectical point

As shown in Table 6, syndrome classification and dialectical point are the tertiary indexes with lower consistency degree. They have been analyzed together because they are related. We can see that the syndrome types *qi* deficiency, phlegm and blood stasis block meridians have the highest frequency (124 cases, 82.7%); *qi* and *yin* deficiency, phlegm-blood stasis and *qi* deficiency, phlegm and heat block meridians respectively are 11 and 7 cases; The three syndrome types above comprise 142 cases (94.67%).

**Table 6:** Syndrome distribution among 150 cases

Syndrome distribution	Syndrome classification	number	percentage (%)
Major Syndrome distribution among 150 cases	Qi deficiency, phlegm and blood stasis	124	82.7
	Qi and yin deficiency, phlegm-blood stasis	11	7.3
	Qi deficiency, phlegm and heat block meridians	7	4.7
Major Syndromes recommended in the CPG among the 150 cases	Heart blood stasis syndrome	140	93.33
	Phlegm-turbidity blockage syndrome	145	96.67
	Qi and yin deficiency	12	8.00

Syndromes recommend by the CPGs as single factors appeared frequently in the 150 cases. However, for each case, a single syndrome factor may not reflect the clinical situation comprehensively.

As shown in Table 7, compared with non-*qi* deficiency, phlegm and blood stasis syndrome, the overall consistency degree of *qi* deficiency, phlegm and blood stasis syndrome was lower, with a statistically significant difference(*P*<0.05).

**Table 7:** Comparison of overall consistency between *qi* deficiency, phlegm and blood stasis syndrome and non-*qi* deficiency, phlegm and blood stasis syndrome

Syndrome classification	number	overall consistency (%)	*z*	*P*
Qi deficiency, phlegm and blood stasis	124	41.30 ± 5.72	−2.35	0.019
Non qi deficiency, phlegm and blood stasis	26	47.15 ± 9.83		

### Application of TCM decoctions

As shown in Table 8, 21 cases refused TCM decoction. Among the 129 cases who used TCM decoction, *Wendan* decoction and *Gualou xiebai* decoction were applied most frequently. Among the main TCM decoctions recommended by CPG, *Gualou xiebai* decoction was applied most frequently. The other TCM decoctions were rarely recommended by the CPGs.

**Table 8:** TCM decoction distribution

	Primary TCM decoction	frequency	percentage (%)
TCM decoction distribution	Wendan decoction	64	42.7
	Gualou xiebai decoction	31	20.7
	Erchen decoction	9	6.0
	Shengmai san	7	4.7
Frequency of TCM decoction recommendations	Gualou xiebai decoction	31	20.7
	Shengmai San	7	4.7
	Shengmai san combined with gualou xiebai decoction	3	2.0
	Taohongsiwu decoction	1	0.7

As shown in Table 9, compared to the group without *Wendan* decoction, the consistency of the group with *Wendan* decoction was low, and the difference was statistically significant(*P*<0.05). we could infer that *Wendan* decoction which was the most frequently applied TCM decoction and not CPG-recommended TCM decoction, was the key factor affecting the consistency.

**Table 9:** Relation between application of *Wendan* decoction and index “TCM decoction” consistency, overall consistency

Main TCM decoction	“TCM decoction” consistency (%)	overall consistency (%)
Used Wendan decoction	29.02 ± 11.75	40.06 ± 3.63
Used other decoctions	44.60 ± 27.03	46.77 ± 8.41
Did not use TCM decoction	0 ± 0	37.33 ± 2.41
*Z*	64.002	47.716
*P*	0.000	0.000

### TCM particular treatment

As shown in Table 10, among 116 cases accepting a particular TCM treatment (Non TCM decoction), “cinnamon and/*or Xiebai* external application” was the most used therapy, followed by the auricular needle therapy recommended by the guidelines.

**Table 10:** TCM Particular treatment application of 150 cases

Particular treatment (Non TCM decoction)	frequency	percentage (%)
Cinnamon and/or Xiebai external application	86	57.3
Auricular needle	24	16.0
Electrical stimulation	4	2.7
Other	2	1.3
Did not use Particular TCM therapies	34	22.7
Total	150	100.0

## Discussion

### The system achieved the preliminary evaluation of the consistency between CPGs and clinical practice

This study established a method to evaluate the consistency of CPGs and clinical practice based on clinical cases. According to the AHP theory, we established a consistency evaluation system for CPGs of TCM. After being tested by clinical cases, this system can be used to quantitatively evaluate the overall consistency of certain guidelines and decipher the inner factors affecting overall consistency. The evaluation results can provide the basis for further revision of CPGs.

Based on the 150 angina cases involved, the result was 42.32% ± 6.94% for the overall consistency of the CPGs of DTCID and clinical practice; the lowest result was 35.21% and the highest was 63.37%. The low results indicate that the CPGs of DTCID for angina were not applied in an ideal fashion. To explore the immanent causes for the low overall consistency, the system can provide some details from the 7 indexes.

The analysis of the consistency degree of each index is as follows:

The consistency of the factor “syndrome classification” was 38.25% ± 4.40%. Among the five syndromes recommended by the CPG, heart blood stasis syndrome and phlegm obstruction syndrome were found in more than 90% of the cases. This only reflected a segment of the syndrome features, as it was difficult to summarize the clinical characteristics of individual specific cases. Among the 150 cases, *qi* deficiency, phlegm and blood stasis were the most common syndrome, accounting for 124 cases (82.7%). This was inconsistent with the CPG recommendations, thus leading to the decrease in overall consistency degree. The syndromes the CPGs recommended most were for one or two syndrome factors, blood stasis, phlegm, *qi* and *yin* deficiency. They failed to comply with the characteristics of the syndromes of clinical cases.

The consistency of the index “TCM decoction” was 31.08% ± 23.64%. There were 42 cases that used the decoction recommended by the CPG. This accounted for 28% of the 150 cases. For the *Gualou xiebai* decoction, there were 34 applications, accounting for 22.67%. Among the 150 cases, the most frequently used main decoction was *Wendan*, which was used in 64 cases (42.7%). To analyze the effect of *Wendan* decoction and consistency, we divided the 150 cases into 3 groups based on application: *Wendan* decoction group, non- *Wendan* decoction group and no decoction group. The consistency of the *Wendan* decoction group was lower than those of the other two groups, and the difference was statistically significant (*p* < 0.05). This shows that the application of *Wendan* decoction in clinical practice was one of the reasons for the decrease in overall consistency.

The consistency of the index “TCM particular treatment” was 7.92 ± 19.13%. The mean value was small and the standard deviation was large, indicating that the consistency degree was lower, but the difference was bigger. TCM particular treatment refers to non-decoction TCM therapies including acupuncture and moxibustion. The CPG recommended therapies including proven decoctions (panax pseudo-ginseng powder, blood and heartache powder) and acupuncture (body acupuncture, moxibustion and ear acupuncture). For various reasons, 34 of the 150 cases did not use non-decoction TCM therapies. Among the 116 cases using TCM therapy, 24 (16%) used the ear acupuncture guidelines recommended. Thus, the consistency degree of this index dropped.

For the indexes “TCM diagnosis” and “Western medicine diagnosis,” since the cases included in the study were in accordance with the diagnostic criteria of the two sets of guidelines, the two indexes were 100% consistent with the evaluation. For “recuperation and prevention,” the guidelines do not refer to preventive measures or other content, so this tertiary index met the consistency degree of 0.

To summarize, the proposals of the CPGs, including syndrome classification, TCM decoction and TCM particular therapy, were infrequently applied in the indexes. This resulted in the low overall consistency.

Furthermore, we found that the system achieves the preliminary evaluation of consistency between CPGs and clinical practice. By analyzing the indexes with low consistency, we also explored the immanent factors which affect the overall consistency. Therefore, we can infer the application of CPGs and make some suggestions on CPGs recension.

### The consistency evaluation is helpful in reflecting on the application of TCM CPGs

The significance and value of a set of guidelines is to effectively guide clinical practice and improve the level of clinical diagnosis and treatment [11]. In clinical practice, the evaluation of the effectiveness of an intervention is the concept of “efficacy” and “effectiveness,” as proposed by the field of Health Technology Assessment (HTA). Efficacy is the application of health technology to a particular health problem in an ideal situation (e.g. well-designed and managed randomized controlled trials) in which the standard for selecting the targets is strict and the study is conducted in a qualified research center. Effectiveness refers to the application of health technology to a certain health problem in general or under normal conditions. For example, in a community hospital, a certain health technology will be applied to a variety of patients by a general practitioner. The result is better when the health technology is applied under strict control conditions or in carefully selected patients, as opposed to under normal conditions. Therefore, the efficacy of an intervention is often better than the effectiveness [12].

Although CPGs are based on evidence, in their effects in clinical practice there still exists a gap between efficacy and effectiveness. A quality evaluation study basing AGREE for first 28 evidence-based TCM CPGs in China [13], of which application evaluation of 23 CPGs scored 0, indicated that application of TCM CPGs should be pay more attention in the CPGs development [14]. Because of this, application evaluation should be regarded as of equal significance to quality.

The system described in this study can quantitatively evaluate the consistency between a certain set of guidelines and clinical practice, while the consistency evaluation reflects the application of CPGs. Therefore, the system was beneficial to application evaluation and explored the underlying causes, so as to gather evidence and ideas for the organization of clinical research and the revision of guidelines.

The system built for this study, and based on TCM CPGs, could expand its range of applications. In general, its methods and steps could be applied to evaluating other CPGs or standards. Additionally, the system’s structure could be modified to fit different objects being evaluated.

### Limitations

TCM clinical doctors who consulted to build the system in this study may not familiar with AHP theory. Therefore, some of the questionnaires did not meet the AHP standards and these data were omitted. Additional AHP training may be necessary for further research.

There also may have been subjectivity influence in this study. When adapting the system in the future, the Delphi method, principle component analysis, factor analysis or other statistical methods might be applicable. Also, the study could not explain the relationship between consistency and clinical effect.

## Conclusions

This study has shown that the system can perform quantitative evaluation of the consistency between TCM CPGs and clinical practice. It also uncovered the indexes which affect the application of TCM CPGs. This may be insightful in the revision of CPGs. We believe that this method of consistency evaluation has the potential to improve the evaluation of CPGs, and will become a valuable instrument in the future.

## Supplementary information

**Additional file 1.** Consultation questionnaire for AHP indexes. This file was the text of Consultation questionnaire. This consultation questionnaire was used to collect the scores according to AHP theory so that the evaluation system was built.

**Additional file 2.** Consultation questionnaire for consistency between CPGs and cases. This file was the text of Consultation questionnaire. This consultation questionnaire was used to collect the scores about the consistency between TCM CPGs and clinical cases according to AHP theory so that the consistency was calculated.

## Data Availability

The results of the study came from two important parts of data. The data used to build the AHP model, which were collected from the questionnaires, would be promised to be available to someone interested after this paper published. The data used to test the model, which were collected from the clinical case system in Guangdong Provincial Hospital of Chinese Medicine, are prohibited to public release by Chinese law in the reason of patients privacy. To strictly testing the model and promote in-depth research, we are looking forward to using clinical data of other hospitals to test the model.

## Abbreviations

AGREE: Appraisal of Guidelines, Research and Evaluation
AHP: Analytic Hierarchy Process
CI: The Inconsistency Indicator of a Pairwise
CPGs of DTCID: The China Association of Chinese Medicine’s Guidelines for Diagnosis and Treatment of Common Internal Diseases in Chinese Medicine Diseases of Modern Medicine
CPGs: Clinical Practice Guidelines
CR: The Random Consistency Ratio
PCI: Percutaneous Coronary Intervention
RI: The Average Random Consistency Indicator
TCM: Traditional Chinese Medicines
