# Supplementary material for: Application evaluation of clinical practice guidelines for traditional Chinese medicine: a clinical analysis based on the analytic hierarchy process
Source: BMC Complement Altern Med. 2019 Oct 22;19:277. doi: 10.1186/s12906-019-2683-5 (PMC6805407; doi:10.1186/s12906-019-2683-5)
Supplement: Supplementary file 1 — Additional file 1. Consultation questionnaire for AHP indexes. This file was the text of Consultation questionnaire. This consultation questionnaire was used to collect the scores according to AHP theory so that the evaluation system was built. [file 12906_2019_2683_MOESM1_ESM.docx]

File 2 Consultation questionnaire for AHP indexes

**Consultation Form of TCM Clinical Guidelines Application Evaluation System**

Name ：

Organization：

Education：□Bachelor below □Bachelor

□Master Degree □Doctor Degree

Gender： Age：

Medical Title：□Chief Physician □Associate Chief Physician

□Doctor-in-charge □Resident Physician

Professional：

Engaged in the clinical field of Chinese medicine： years

Dear Expert：

We have been performing the study which aims to evaluate the consistency between Clinical Practice Guidelines (CPGs) of TCM and clinical practice. According to Analytic Hierarchy Process (AHP), main contents of TCM CPGs have been broken and a 3-level AHP construction was built.

Now asking for your opinion as below：

1.Suggestion for 3-level AHP construction

2.Score of the pairwise comparisons in the indexes

Sincerely thank you for your help.

Guangdong provincial hospital of Chinese Medicine 2014-5-12

**Part I Suggestion for 3-level AHP construction**

**
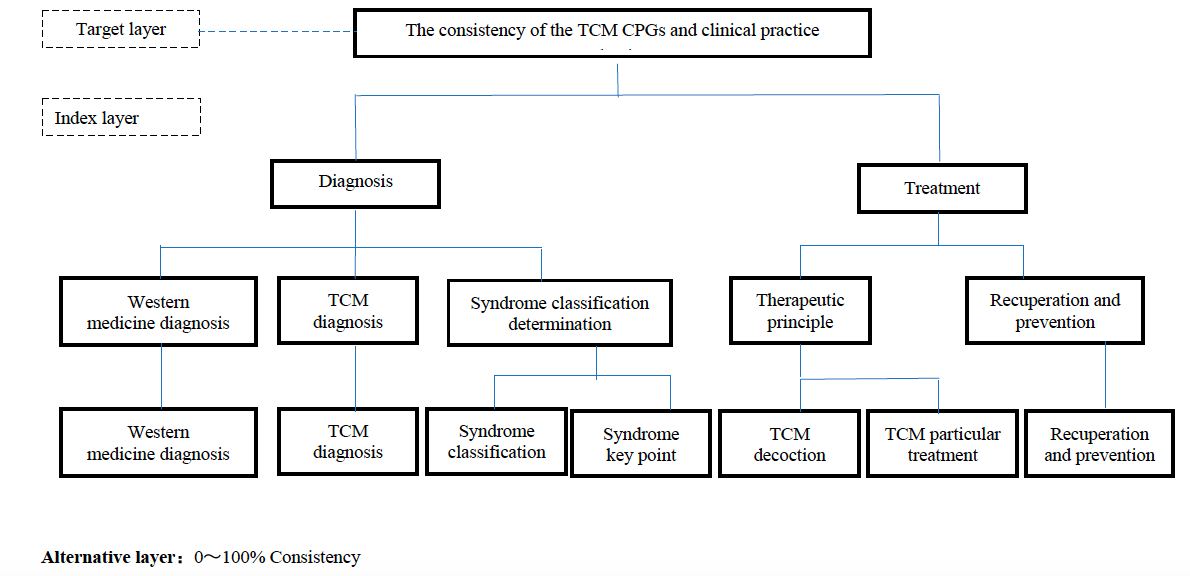
**

According AHP theory，TCM CPGs have been broken into 3 levels and the construction of evaluation system has been built.

Is it reasonable or needed revisions？

**Part II** **Comparison of the importance of each evaluation indicator**

**This is Example for pairwise comparisons of the importance of each indexes.**

**Please compare the importance between the indexes of Diagnosis and Treatment**

| **Score** | **9** | **8** | **7** | **6** | **5** | **4** | **3** | **2** | **1** | **2** | **3** | **4** | **5** | **6** | **7** | **8** | **9** | **Score** |
| --- | --- | --- | --- | --- | --- | --- | --- | --- | --- | --- | --- | --- | --- | --- | --- | --- | --- | --- |
| **Diagnosis** | extremely important |  | much more important |  | more important |  | slightly more important |  | same important |  | slightly more important |  | more important |  | much more important |  | extremely important | **Treatment** |

**■ ★**

**If you recognize Diagnosis is slightly more important than Treatment，you should choose Score 3（place marked ■）.**

**if you recognize Treatment is slightly more important than Diagnosis，you should choose Score 3（the place marked ★）,which would be changed to 1/3 according to AHP Saaty method.**

**(2, 4, 6, 8) was the intermediate value of the two adjacent degrees and was used when necessary.**

**1.The pairwise comparison for the primary indexes**

**Please compare the relative importance in the CPGs between the indexes of Diagnosis and Treatment（Score using √）**

| **Score** | **9** | **8** | **7** | **6** | **5** | **4** | **3** | **2** | **1** | **2** | **3** | **4** | **5** | **6** | **7** | **8** | **9** | **Score** |
| --- | --- | --- | --- | --- | --- | --- | --- | --- | --- | --- | --- | --- | --- | --- | --- | --- | --- | --- |
| **Diagnosis** | extremely important |  | much more important |  | more important |  | slightly more important |  | same important |  | slightly more important |  | more important |  | much more important |  | extremely important | **Treatment** |

**2.The pairwise comparison for the secondary indexes**

**2.1 Please compare the relative importance in the CPGs between the indexes of** **TCM diagnosis and** **Western medicine diagnosis（Score using √）.**

| **Score** | **9** | **8** | **7** | **6** | **5** | **4** | **3** | **2** | **1** | **2** | **3** | **4** | **5** | **6** | **7** | **8** | **9** | **Score** |
| --- | --- | --- | --- | --- | --- | --- | --- | --- | --- | --- | --- | --- | --- | --- | --- | --- | --- | --- |
| **TCM diagnosis** | extremely important |  | much more important |  | more important |  | slightly more important |  | same important |  | slightly more important |  | more important |  | much more important |  | extremely important | **Western medicine diagnosis** |

**2.2 Please compare the relative importance in the CPGs between the indexes of TCM diagnosis and** **Syndrome differentiation**  **determination（Score using √）.**

| **Score** | **9** | **8** | **7** | **6** | **5** | **4** | **3** | **2** | **1** | **2** | **3** | **4** | **5** | **6** | **7** | **8** | **9** | **Score** |  |  |
| --- | --- | --- | --- | --- | --- | --- | --- | --- | --- | --- | --- | --- | --- | --- | --- | --- | --- | --- | --- | --- |
| **TCM diagnosis** | | extremely important |  | much more important |  | more important |  | slightly more important |  | same important |  | slightly more important |  | more important |  | much more important |  | extremely important | **Syndrome differentiation**  **determination** | |

**2.3 Please compare the relative importance in the CPGs between the indexes of** **Western medicine diagnosis and Syndrome differentiation** **determination（Score using √）.**

| **Score** | **9** | **8** | **7** | **6** | **5** | **4** | **3** | **2** | **1** | **2** | **3** | **4** | **5** | **6** | **7** | **8** | **9** | **Score** |  |  |
| --- | --- | --- | --- | --- | --- | --- | --- | --- | --- | --- | --- | --- | --- | --- | --- | --- | --- | --- | --- | --- |
| **Western medicine diagnosis** | | extremely important |  | much more important |  | more important |  | slightly more important |  | same important |  | slightly more important |  | more important |  | much more important |  | extremely important | **Syndrome differentiation**  **determination** | |

**2.4 Please compare the relative importance in the CPGs between the indexes of therapeutic principle and** **Recuperation and prevention（Score using √）.**

| **Score** | **9** | **8** | **7** | **6** | **5** | **4** | **3** | **2** | **1** | **2** | **3** | **4** | **5** | **6** | **7** | **8** | **9** | **Score** |  |  |
| --- | --- | --- | --- | --- | --- | --- | --- | --- | --- | --- | --- | --- | --- | --- | --- | --- | --- | --- | --- | --- |
| **Therapeutic principle** | | extremely important |  | much more important |  | more important |  | slightly more important |  | same important |  | slightly more important |  | more important |  | much more important |  | extremely important | **Recuperation and prevention** | |

**3.The pairwise comparison for the tertiary indexes**

**3.1 Please compare the relative importance in the CPGs between the indexes of Syndrome classification and Syndrome key point（Score using √）.**

| **Score** | **9** | **8** | **7** | **6** | **5** | **4** | **3** | **2** | **1** | **2** | **3** | **4** | **5** | **6** | **7** | **8** | **9** | **Score** |  |
| --- | --- | --- | --- | --- | --- | --- | --- | --- | --- | --- | --- | --- | --- | --- | --- | --- | --- | --- | --- |
| **Syndrome classification** | | extremely important |  | much more important |  | more important |  | slightly more important |  | same important |  | slightly more important |  | more important |  | much more important |  | extremely important | **Syndrome**  **key point** |

**3.2 Please compare the relative importance in the CPGs between the indexes of TCM decoction and TCM particular treatment（Score using √）**

| **Score** | **9** | **8** | **7** | **6** | **5** | **4** | **3** | **2** | **1** | **2** | **3** | **4** | **5** | **6** | **7** | **8** | **9** | **Score** |  |
| --- | --- | --- | --- | --- | --- | --- | --- | --- | --- | --- | --- | --- | --- | --- | --- | --- | --- | --- | --- |
| **TCM decoction** | | extremely important |  | much more important |  | more important |  | slightly more important |  | same important |  | slightly more important |  | more important |  | much more important |  | extremely important | **TCM particular**  **treatment** |
