# Supplementary material for: Application evaluation of clinical practice guidelines for traditional Chinese medicine: a clinical analysis based on the analytic hierarchy process
Source: BMC Complement Altern Med. 2019 Oct 22;19:277. doi: 10.1186/s12906-019-2683-5 (PMC6805407; doi:10.1186/s12906-019-2683-5)
Supplement: Supplementary file 2 — Additional file 2. Consultation questionnaire for consistency between CPGs and cases. This file was the text of Consultation questionnaire. This consultation questionnaire was used to collect the scores about the consistency between TCM CPGs and clinical cases according to AHP theory so that the consistency was calculated. [file 12906_2019_2683_MOESM2_ESM.docx]

File 3 Consultation questionnaire for consistency between CPGs and cases

**The Cases Report Form of TCM Clinical Guidelines Application Evaluation based the AHP System**

**Basic Information of the patient**

**Name：**

**Age：**

**Gender：Male □ Female □**

**Medical record number：**

**Hospital stay： Days**

**PCI operation：□PCI surgery before hospitalization**

**□PCI surgery before hospitalization**

**□no PCI surgery**

**Evaluator（signature）：**

**Operation Description**

According to clinical practice，please evaluate the consistency between CPGs of TCM and clinical cases in tertiary indexes and score using the Saaty weighting method.

| **Consistency（100%）** | **100%** | **～** | **87.5%** | **～** | **75%** | **～** | **62.5%** | **～** | **50%** | **～** | **37.5%** | **～** | **25%** | **～** | **12.5%** | **～** | **0%** | **Consistency（100%）** |
| --- | --- | --- | --- | --- | --- | --- | --- | --- | --- | --- | --- | --- | --- | --- | --- | --- | --- | --- |
| **Saaty Score** | 9 | 8 | 7 | 6 | 5 | 4 | 3 | 2 | 1 | 2 | 3 | 4 | 5 | 6 | 7 | 8 | 9 | **Saaty Score** |
| **Fully**  **Consistency** |  |  |  |  |  |  |  |  | **√** |  |  |  |  |  |  |  |  | **Non**  **Consistency** |

**Example:**

If the evaluators suggested the consistency between CPGs of TCM and clinical cases in the tertiary index be 50%，they should score 50% using √. Result from the score of each index, the system would calculate the total consistency of each patient.

**1.Diagnosis**

**1.1 TCM Diagnosis**

**TCM Diagnosis of** **the patient：**

**According to this patient, please compare the** **consistency between the CPG and clinical practice.**

| **Consistency（100%）** | **100%** | **～** | **87.5%** | **～** | **75%** | **～** | **62.5%** | **～** | **50%** | **～** | **37.5%** | **～** | **25%** | **～** | **12.5%** | **～** | **0%** | **Consistency（100%）** |
| --- | --- | --- | --- | --- | --- | --- | --- | --- | --- | --- | --- | --- | --- | --- | --- | --- | --- | --- |
| **Saaty Score** | 9 | 8 | 7 | 6 | 5 | 4 | 3 | 2 | 1 | 2 | 3 | 4 | 5 | 6 | 7 | 8 | 9 | **Saaty Score** |
| **Fully Consistency** |  |  |  |  |  |  |  |  |  |  |  |  |  |  |  |  |  | **Non**  **Consistency** |

**Reasons for Non-consistency：**

**Suggestions for CPG revision：**

**1.2 Western medicine diagnosis**

**Western medicine diagnosis of the patient：**

**According to this patient, please compare the consistency between the CPG and clinical practice.**

| **Consistency（100%）** | **100%** | **～** | **87.5%** | **～** | **75%** | **～** | **62.5%** | **～** | **50%** | **～** | **37.5%** | **～** | **25%** | **～** | **12.5%** | **～** | **0%** | **Consistency（100%）** |
| --- | --- | --- | --- | --- | --- | --- | --- | --- | --- | --- | --- | --- | --- | --- | --- | --- | --- | --- |
| **Saaty Score** | 9 | 8 | 7 | 6 | 5 | 4 | 3 | 2 | 1 | 2 | 3 | 4 | 5 | 6 | 7 | 8 | 9 | **Saaty Score** |
| **Fully Consistency** |  |  |  |  |  |  |  |  |  |  |  |  |  |  |  |  |  | **Non**  **Consistency** |

**Reasons for Non-consistency：**

**Suggestions for CPG revision：**

**1.3 Syndrome classification determination**

**1.3.1 Syndrome classification**

**Syndrome classification of the patient：**

**According to this patient, please compare the consistency between the CPG and clinical practice.**

| **Consistency（100%）** | **100%** | **～** | **87.5%** | **～** | **75%** | **～** | **62.5%** | **～** | **50%** | **～** | **37.5%** | **～** | **25%** | **～** | **12.5%** | **～** | **0%** | **Consistency（100%）** |
| --- | --- | --- | --- | --- | --- | --- | --- | --- | --- | --- | --- | --- | --- | --- | --- | --- | --- | --- |
| **SaatyScore** | 9 | 8 | 7 | 6 | 5 | 4 | 3 | 2 | 1 | 2 | 3 | 4 | 5 | 6 | 7 | 8 | 9 | **SaatyScore** |
| **Fully Consistency** |  |  |  |  |  |  |  |  |  |  |  |  |  |  |  |  |  | **Non**  **Consistency** |

**Reasons for Non-consistency：**

**Suggestions for CPG revision：**

**1.3.2 Syndrome key point**

**Syndrome key point of the patient：**

**According to this patient, please compare the consistency between the CPG and clinical practice.**

| **Consistency（100%）** | **100%** | **～** | **87.5%** | **～** | **75%** | **～** | **62.5%** | **～** | **50%** | **～** | **37.5%** | **～** | **25%** | **～** | **12.5%** | **～** | **0%** | **Consistency（100%）** |
| --- | --- | --- | --- | --- | --- | --- | --- | --- | --- | --- | --- | --- | --- | --- | --- | --- | --- | --- |
| **SaatyScore** | 9 | 8 | 7 | 6 | 5 | 4 | 3 | 2 | 1 | 2 | 3 | 4 | 5 | 6 | 7 | 8 | 9 | **SaatyScore** |
| **Fully Consistency** |  |  |  |  |  |  |  |  |  |  |  |  |  |  |  |  |  | **Non**  **Consistency** |

**Reasons for Non-consistency：**

**Suggestions for CPG revision：**

**2.Treatment**

**2.1 Therapeutic principle**

**2.1.1 TCM decoction of the patient：**

**According to this patient, please compare the consistency between the CPG and clinical practice.**

| **Consistency（100%）** | **100%** | **～** | **87.5%** | **～** | **75%** | **～** | **62.5%** | **～** | **50%** | **～** | **37.5%** | **～** | **25%** | **～** | **12.5%** | **～** | **0%** | **Consistency（100%）** |
| --- | --- | --- | --- | --- | --- | --- | --- | --- | --- | --- | --- | --- | --- | --- | --- | --- | --- | --- |
| **SaatyScore** | 9 | 8 | 7 | 6 | 5 | 4 | 3 | 2 | 1 | 2 | 3 | 4 | 5 | 6 | 7 | 8 | 9 | **SaatyScore** |
| **Fully Consistency** |  |  |  |  |  |  |  |  |  |  |  |  |  |  |  |  |  | **Non**  **Consistency** |

**Reasons for Non-consistency：**

**Suggestions for CPG revision：**

**2.1.2 TCM particular treatment**

**TCM particular treatment of the patient：**

**According to this patient, please compare the consistency between the CPG and clinical practice.**

| **Consistency（100%）** | **100%** | **～** | **87.5%** | **～** | **75%** | **～** | **62.5%** | **～** | **50%** | **～** | **37.5%** | **～** | **25%** | **～** | **12.5%** | **～** | **0%** | **Consistency（100%）** |
| --- | --- | --- | --- | --- | --- | --- | --- | --- | --- | --- | --- | --- | --- | --- | --- | --- | --- | --- |
| **SaatyScore** | 9 | 8 | 7 | 6 | 5 | 4 | 3 | 2 | 1 | 2 | 3 | 4 | 5 | 6 | 7 | 8 | 9 | **SaatyScore** |
| **Fully Consistency** |  |  |  |  |  |  |  |  |  |  |  |  |  |  |  |  |  | **Non**  **Consistency** |

**Reasons for Non-consistency：**

**Suggestions for CPG revision：**

**2.2** **Recuperation and prevention**

**Recuperation and prevention of the patient：**

**According to this patient, please compare the consistency between the CPG and clinical practice.**

| **Consistency（100%）** | **100%** | **～** | **87.5%** | **～** | **75%** | **～** | **62.5%** | **～** | **50%** | **～** | **37.5%** | **～** | **25%** | **～** | **12.5%** | **～** | **0%** | **Consistency（100%）** |
| --- | --- | --- | --- | --- | --- | --- | --- | --- | --- | --- | --- | --- | --- | --- | --- | --- | --- | --- |
| **SaatyScore** | 9 | 8 | 7 | 6 | 5 | 4 | 3 | 2 | 1 | 2 | 3 | 4 | 5 | 6 | 7 | 8 | 9 | **SaatyScore** |
| **Fully Consistency** |  |  |  |  |  |  |  |  |  |  |  |  |  |  |  |  |  | **Non**  **Consistency** |

**Reasons for Non-consistency：**

**Suggestions for CPG revision：**

**3.Evaluation of the clinical effects.**

**The Canadian Cardiovascular Society (CCS) angina severity classification is applied to evaluate the clinical effects.**

Classification before Hospitalization：

Classification after Hospitalization：

The Canadian Cardiovascular Society (CCS) angina severity classification

Class 1: Angina with strenuous Exercise

Class 2: Angina with moderate exertion

Class 3: Angina with mild exertion

Walking 1-2 level blocks at normal pace

Climbing 1 flight of stairs at normal pace

Class 4: Angina at any level of physical exertion
